# The CXCL16-CXCR6 axis in glioblastoma modulates T-cell activity in a spatiotemporal context

**DOI:** 10.3389/fimmu.2023.1331287

**Published:** 2024-01-17

**Authors:** Tzu-Yi Chia, Leah K. Billingham, Lauren Boland, Joshua L. Katz, Victor A. Arrieta, Jack Shireman, Aurora-Lopez Rosas, Susan L. DeLay, Kaylee Zillinger, Yuheng Geng, Jeandre Kruger, Caylee Silvers, Hanxiang Wang, Gustavo Ignacio Vazquez Cervantes, David Hou, Si Wang, Hanxiao Wan, Adam Sonabend, Peng Zhang, Catalina Lee-Chang, Jason Miska

**Affiliations:** ^1^ Department of Neurological Surgery, Feinberg School of Medicine, Northwestern University, Chicago, IL, United States; ^2^ Malnati Brain Tumor Institute of the Robert H. Lurie Comprehensive Cancer Center, Feinberg School of Medicine, Northwestern University, Chicago, IL, United States; ^3^ Stanley Manne Children’s Research Institute, Ann & Robert H. Lurie Children’s Hospital, Chicago, IL, United States; ^4^ Department of Neurosurgery, University of Wisconsin School of Medicine & Public Health, UW Carbone Cancer Center, Madison, WI, United States

**Keywords:** glioblastoma, CXCL16, CXCR6, immunotherapy, myeloid cells

## Abstract

**Introduction:**

Glioblastoma multiforme (GBM) pathobiology is characterized by its significant induction of immunosuppression within the tumor microenvironment, predominantly mediated by immunosuppressive tumor-associated myeloid cells (TAMCs). Myeloid cells play a pivotal role in shaping the GBM microenvironment and influencing immune responses, with direct interactions with effector immune cells critically impacting these processes.

**Methods:**

Our study investigates the role of the CXCR6/CXCL16 axis in T-cell myeloid interactions within GBM tissues. We examined the surface expression of CXCL16, revealing its limitation to TAMCs, while microglia release CXCL16 as a cytokine. The study explores how these distinct expression patterns affect T-cell engagement, focusing on the consequences for T-cell function within the tumor environment. Additionally, we assessed the significance of CXCR6 expression in T-cell activation and the initial migration to tumor tissues.

**Results:**

Our data demonstrates that CXCL16 surface expression on TAMCs results in predominant T-cell engagement with these cells, leading to impaired T-cell function within the tumor environment. Conversely, our findings highlight the essential role of CXCR6 expression in facilitating T-cell activation and initial migration to tumor tissues. The CXCL16-CXCR6 axis exhibits dualistic characteristics, facilitating the early stages of the T-cell immune response and promoting T-cell infiltration into tumors. However, once inside the tumor, this axis contributes to immunosuppression.

**Discussion:**

The dual nature of the CXCL16-CXCR6 axis underscores its potential as a therapeutic target in GBM. However, our results emphasize the importance of carefully considering the timing and context of intervention. While targeting this axis holds promise in combating GBM, the complex interplay between TAMCs, microglia, and T cells suggests that intervention strategies need to be tailored to optimize the balance between promoting antitumor immunity and preventing immunosuppression within the dynamic tumor microenvironment.

## Introduction

Glioblastoma, the most aggressive and malignant form of primary brain cancer, presents a daunting challenge in the field of oncology due to its highly infiltrative nature and resistance to conventional therapies (1, 2). An emerging facet of glioblastoma pathobiology is its profound ability to induce immunosuppression within the tumor microenvironment (3). This immunosuppressive milieu is characterized by the recruitment of immunosuppressive tumor-associated myeloid cells (TAMCs), which are, by far, the most predominant cellular infiltrates in GBM (4). TAMC is an umbrella term for all infiltrating tumor-associated macrophages (TAMs), myeloid-derived suppressor cells (MDSCs), and other monocytes in tumors that are not microglia (phenotypically referred to as CD45^+^CD11b^+^). This population is predominantly composed of monocytic-MDSC populations but contains several phenotypically diverse populations (5–9). TAMCs play a pivotal role in shaping the GBM tumor microenvironment by both promoting tumor growth and inhibiting antitumor immune responses (7, 9, 10).

Whether fostering antitumor immunity or promoting immunosuppression, an important aspect of myeloid cell biology, is the need for direct physical interactions with effector immune cells (11, 12). In the context of promoting antitumor immune responses, myeloid cells, such as dendritic cells and macrophages, initiate these interactions by presenting tumor antigens to T cells, thereby activating cytotoxic immune responses against cancer cells (13–15). Conversely, in the promotion of immunosuppression, TAMCs (including dendritic cells) often establish contact with T cells, inhibiting their effector functions and dampening the immune response against tumors (12, 16, 17). Therefore, crosstalk between myeloid cells and immune cells at the cellular level is a crucial determinant of the balance between antitumor immunity and immunosuppression within the tumor microenvironment, suggesting that this crosstalk is a focal point for therapeutic interventions in cancer immunotherapy.

Despite the essential nature of immune cell contact in affecting immunosuppression in different tumors, the drivers of these interactions in GBM are not well described. Analysis of GBM in the clinical setting has suggested that the CXCR6/CXCL16 axis is one of the most prominent chemokine/receptor interactions between myeloid cells and GBM (18). While the CXCL16 chemokine was initially identified as a scavenger receptor for oxidized low-density lipoprotein (oxLDL) (19), it was later recognized for its function as both a transmembrane receptor and a soluble chemoattractant. Its cognate receptor, CXCR6, is expressed on activated CD8^+^ T cells and other tissues in the body, and the interaction between CXCL16 and CXCR6 promotes firm cellular contact and adhesion (20).

The CXCL16-CXCR6 axis is recognized for its ability to regulate immune responses. However, its involvement in either enhancing or suppressing antitumor immunity is unclear due to findings indicating its potential for both functions (21–25). In this study, we aimed to understand the nature of these interactions in GBM and how they relate to antitumor immunity. Initially, we observed that CXCL16 surface expression is exclusive to TAMCs, while microglial expression results in the secretion of this cytokine. Importantly, we were able to show that this compartmentalization occurs in the seminal Neftel et al. human GBM datasets (26). Subsequently, we determined that the CXCR6-CXCL16 axis facilitates T-cell/TAMC interactions both *in vivo* and *in vitro*. Intriguingly, our data reveal distinct effects in different compartments, potentially revealing discrepancies in the literature. Specifically, our findings suggest that CXCR6 expression is crucial for T-cell activation and migration into the brain; however, within the tumor microenvironment, its expression appears to induce T-cell dysfunction. Consequently, our results underscore the dualistic nature of this axis in influencing antitumor immunity.

## Methods

### Mice

C57BL/6, CXCR6^0/0^BL/6, Rag^0/0^ BL/6, and CD45.1^+/+^BL/6 mice were obtained from The Jackson Laboratory (Bar Harbor, ME) and bred for use at the Center for Comparative Medicine at Northwestern Feinberg School of Medicine. The mice were housed in a conventional barrier facility where they always had access to food and water and a 12-hour light and 12-hour dark cycle. We used 6- to 8-week-old mice for all the experiments, and the mice were matched in terms of both age and sex. All the experiments involving mouse studies were approved by Northwestern Institutional Animal Care and Use Committee, with the study approval number IS00017401.

### Cell lines and tumor implantation

The CT-2A cell line, a murine syngeneic glioma cell line, was obtained from Sigma–Aldrich/Millipore. The cells were cultured in Dulbecco’s modified Eagle’s medium (DMEM; Corning) supplemented with 10% fetal bovine serum (FBS; HyClone) and penicillin−streptomycin at 37°C with 5% CO_2_. For injections, CT-2A tumor cells were lifted with trypsin-EDTA (Corning), washed with phosphate-buffered saline (PBS), and resuspended at a concentration of 1 × 10^5^ cells/2.5 μL. Mice aged 6-8 weeks were intracranially implanted at a depth of 3 mm with 1 x 10^5^ tumor cells per 2.5 μL of PBS using a stereotactic apparatus. For cannula injection, we injected 5 x 10^4^ tumor cells/2.5 μL. For the survival experiment involving anti-PD-1 therapy or combination therapy, we intraperitoneally injected the cells at a dose of 10 mg/kg every three days.

### *In vitro* T-cell/TAMC generation and expansion

Purified T cells were isolated from single-cell suspensions of mouse spleens using an EasySep Mouse T-cell isolation kit (STEMCELL), cultured in complete RPMI, stimulated with Dynabeads (Invitrogen, ratio of 1:3 beads/T cell), and expanded with 2000 U/mL recombinant IL-2 (PeproTech) for 72 hours (37°C, 5% CO_2_).

Bone marrow progenitor cells were obtained from the femurs and tibias of C57BL/6 mice. After red blood cell lysis and 70 µm filtering, the cells were resuspended in complete RPMI medium and cultured with MCSF (40 ng/mL, PeproTech) at a density of 2.5x10^5^/mL in 24-well plates. The old media was replaced with 50% fresh cRPMI and 50% sterile-filtered CT2A glioma cell culture medium supplemented with MCSF (1 µl/mL) after 3 days of culture. The cells were then collected for further 3 days of culture.

### Mouse sample preparation for scRNA-seq

To prepare murine cells for single-cell RNA sequencing (scRNA-seq), CT-2A tumors were subjected to microdissection following 14 days of tumor growth. Dissociation into a single-cell suspension was achieved using an adult brain dissociation kit (Miltenyi) following the manufacturer’s protocol. Subsequently, CD45^+^ cells, which are indicative of immune cells, were isolated through magnetic bead-based separation (Miltenyi). A mixture of CD45^+^ and CD45^-^ cells was then created at a ratio of 10:1 to capture the transcriptional profiles of both immune cells (CD45^+^) and tumor cells (CD45^-^). The prepared samples were then submitted to the NUSEQ core.

For single-cell library preparation and sequencing, the procedures were conducted at the Northwestern University NUseq facility core with support from the NIH Grant (1S10OD025120). Cell number and viability were assessed using a Nexcelom Cellometer Auto2000 with AOPI fluorescence staining. Sixteen thousand cells were loaded into a Chromium Controller (10X Genomics, PN-120223) on a Chromium Next GEM Chip K (10X Genomics, PN-1000127) and processed to generate single-cell gel beads in the emulsion (GEM) according to the manufacturer’s protocol. The cDNA and library were generated using the Chromium Next GEM Single Cell 5’ Reagent Kit v2 (10X Genomics, PN-1000283) following the manufacturer’s manual. Additionally, mouse T-cell and B-cell V(D)J libraries were constructed using the Chromium Single-cell Mouse TCR and BCR Amplification Kit (10X Genomics, PN-1000252, and PN-1000255).

The multiplexed libraries were combined and sequenced on an Illumina HiSeq 4000 sequencer with 50 paired-end kits utilizing a read length of 28 bp for Read1 (cell barcode and UMI) and 91 bp for Read2 (transcript). The targeted sequencing depths for the gene expression, mouse T cell, and B-cell V(D)J libraries were set at 20,000, 5,000, and 5,000 reads per cell, respectively.

### scRNA-seq analysis

For the human data shown, the Neftel et al. datasets (26) were used for the initial analysis. Single-cell analysis was conducted on both human and mouse samples via the 10x chromium platform, and the reads were processed using CellRanger. Seurat v5 was used for downstream analysis with default parameters as per recommended usage. Clustering was performed using UMAP dimensional reduction after PCA and validation of variation with elbow plots and JackStraw testing. Cell type assignment was conducted using the unbiased SingleR package, where each cell was annotated according to the alignment to the mouse gene expression reference provided by Celldex. All visualizations were performed within Seurat.

For dot plots, the relevant figures depict two scales: one determines the dot size, representing the quantity of cells within the subgroup, and the other dictates the color, indicating the expression level of cells within the subgroup. The expression values are scaled proportionally within the subset of cells isolated within the Seurat cohort. To clarify, the figure serves as an intuitive means of visualizing how feature expression changes across different identity classes (clusters). Dot size encodes the percentage of cells within a class, while color encodes the average expression level across all cells within a class, with blue denoting high expression.

### Multiplex immunofluorescence staining

N=22 sections, 5 μm thick, were derived from formalin-fixed, paraffin-embedded GBM samples from both primary and recurrent GBM patients. Patient characteristics can be found in **Supplementary Table 1**. These slides were deparaffinized with BOND dewaxax solution and then subjected to heat-induced epitope retrieval using either BOND epitope retrieval solution (pH 6) or pH 9 EDTA buffer for a 20-minute period. Subsequently, the slides were blocked with peroxide and protein. The primary antibodies, which were diluted using the 1x Opal Antibody diluent/block solution, were paired with the respective Opal dyes: 1) TMEM119 (cat. HPA051870, Sigma−Aldrich, 1:200 dilution) with Opal 520 (1:100 dilution); 2) CXCL16 (cat. MA5-27845, clone GT516, Invitrogen, 1:1000 dilution) with Opal 570 (1:200 dilution); 3) CXCR6 (cat. ab8023, Abcam, dilution 1:500) with Opal 540 (1:200 dilution); 4) CD163 (cat. ab213612, clone EPR19518, Abcam, 1:600 dilution) with Opal 650 (1:200 dilution); 5) CD3 (cat. PA0553, clone LN10, Leica, RTU); and 6) CD8 (cat. PA0183, clone 4B11, Leica, RTU). The multiplex staining process involved several cycles, each of which included heat-induced epitope retrieval, protein blocking, epitope labeling, and signal amplification. After all the markers were stained, the slides were counterstained with spectral DAPI and finally sealed with Prolong Diamond Antifade Mountant.

### Multispectral imaging and analysis

The Vectra 3 Automated Quantitative Pathology Imaging System from Akoya Biosciences was used to obtain multispectral images (MSIs). Initially, the entire slide was imaged, adjusting the focus and signal intensity automatically. High-intensity imaging (MSI) was then performed on the tumor sections, which were marked by a qualified neuropathologist at a magnification of 20x. For the analysis of the acquired MS image, a spectral library for all Opal dyes was constructed. This library aided in spectral unmixing, making it possible to differentiate weak signals and overlaps from the background, allowing for clear visualization of each marker via inForm Tissue Finder software. With this software, the adaptive cell segmentation feature pinpoints the nuclei of the analyzed cells and demarcates the nuclear and cytoplasmic sections of each cell. With the help of a training algorithm in inForm, each marker was identified, and all cells were categorized into specific phenotypes. The data exported from inForm were processed in R software using the Phenoptr and PhenoptrReports packages to merge the data and form consolidated files for every tumor sample. These consolidated files had both single and double phenotypes and were subsequently used for further quantification and spatial analysis through the Phenoptr R addin. Finally, the consolidated files were analyzed via Phenoptr to determine the cell density for each marker.

### Immunophenotyping

Before flow cytometry analysis, single-cell suspensions were incubated with the fixable viability dye eFluor780 for 20 minutes, blocked for 5 minutes, and stained with the following antibodies for 15 minutes, except for anti-CXCL16 PE, for 30 minutes at 37°C on ice for flow cytometric analysis. For cytokine staining, the cells were preincubated with Cell Stimulation Cocktail plus protein transport inhibitors (Invitrogen) for 4 hours prior to fixation and intracellular staining. The cells were fixed and permeabilized for intracellular staining utilizing an intracellular fixation and permeabilization buffer set. (Invitrogen)

For *in vivo* studies, the following antibodies were used: Anti-CXCR6 PE, anti-CD4 BUV396, anti-CD44 PerCP-Cy5.5, anti-Foxp3 Pacific Blue, anti-CD8 BV605, anti-CD62L BV711, anti-LAG3 APC, anti-CD3 AF700, the fixable viability dye eFluor780. nti-PD1 PE, and anti-Tim3 PeCy7 as the exhaustion panel. The cytokine panel used was composed of: anti-CD4 BUV396, anti-CD44 PerCP-Cy5.5, anti-TNFα BV421, anti-CD45 AmCyan, anti-CD8 BV605, anti-CD11b BV711, anti-GzmB APC, anti-IFNg AF700, the fixable viability dye eFluor780, and anti-interleukin-17 PeCy7. Anti-Ly6G PerCP-Cy5.5, anti-CD45 AmCyan, anti-CD11b BV711, anti-Ly6C AF700, the fixable viability dye eFluor780, and anti-PDL1 PECy7 were used as the myeloid panel. Please refer to **Supplementary Table 2** for a list of all antibodies, conjugates, sources, clones, and RRIDs.

### *In vitro* adhesion assay

Before the adhesion assay, wild-type and CXCR6 knockout CD8^+^ T cells were stained with red and green cell trackers, respectively. The labeled CD8^+^ T cells and TAMCs were plated at 2:1 and 4:1 dilutions, respectively, in the same slide chamber at 37°C with 5% CO2. After 2 hours of coculture, the nonbinding cells were collected by pipetting gently with PBS twice. A Leica DMi8 microscope was used to visualize the remaining cells.

### Quantification and statistical analysis

The statistical significance of differences between two groups was assessed by a two-tailed unpaired Student’s t test for individual comparisons. For comparisons involving three or more groups, we conducted one-way analysis of variance (ANOVA) followed by Tukey’s *post hoc* test. K−M survival curves were generated, and the significance of the differences in *in vivo* survival was determined through the log-rank test. P values were calculated using Prism software (GraphPad, San Diego, CA) as specified in the figure legends. The error bars in all the figures represent the means ± SEMs.

## Results

### The CXCL16/CXCR6 axis is enhanced in the GBM tumor microenvironment

**Figure 1:**
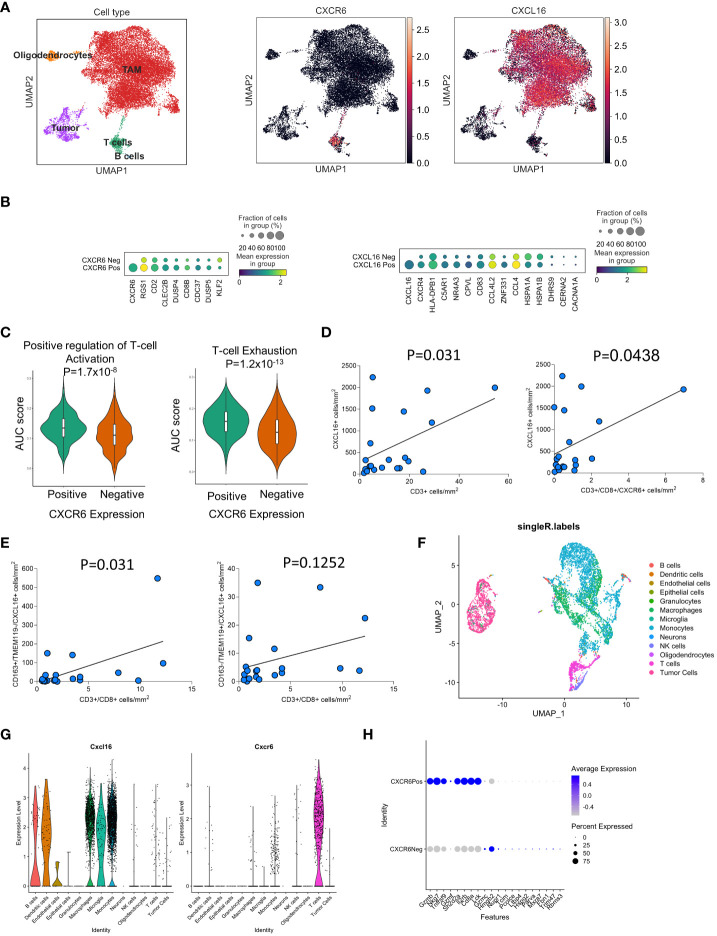
The CXCL16/CXCR6 axis is partitioned into myeloid/T-cell populations in GBM based on scRNAseq analyses. In **(A–C)**, the expression of CXCL16 and CXCR6 was examined in the Neftel et al. human scRNA-seq GBM datasets. In (**B –** *Left panel*), the DEGs between CXCR6^+^ and CXCR6^-^ within the T-cell cluster (defined by CD3ϵ expression) in GBM are shown. In (**B**, *right panel*), the differential expression of genes in the CXCL16^+^ *vs*. CXCL16^-^ clusters within the myeloid cluster (defined by CD14 expression) in GBM. In **(C)**, the area under the curve (AUC) for gene sets associated with T-cell activation and exhaustion was compared between CXCR6^+^ and CXCR6^-^ T cells in human GBM. In **(D, E)**, multiplex immunohistochemistry was performed on 22 newly diagnosed and recurrent GBM samples. The correlation of T-cell infiltration is based on the expression of CXCL16^+^ cells in **(D)** and immunosuppressive/microglia in **(E)**. In **(F–H)**, scRNA analysis of CT-2A tumor-bearing mice is shown. In **(F)**, UMAP projection and single R annotation of cellular infiltrates in CT2A tumors are shown. In **(G)**, the expression of CXCL16 and CXCR6 was among the genes in these clusters. In **(H)**, the DEGs between CXCR6+ and CXCR6- T cells in CT-2A tumors are shown. In **(D, E)**, for 22 patients, both newly diagnosed and recurrent tumors were analyzed via simple linear regression. P values for all comparisons are directly stated in the figures.

We began by characterizing the potential interaction between T-cell cytokines and myeloid cells in the GBM TME. By analyzing previously published datasets of human primary GBM tissue from Neftel et al. (26), we found that the expression of CXCR6 is restricted to T cells, while the expression of its ligand CXCL16 is broadly expressed across all myeloid subsets (**Figure 1A**). Importantly, previous work has shown that in newly diagnosed GBM, this is the only significant T-cell-myeloid interaction identified (20). Examination of the functional status of T cells in human GBM revealed that CXCR6-positive T cells are more likely to be activated and exhausted than CXCR6- cells are. (**Figure 1C**). CXCR6 expression is strongly correlated with CD8α expression, which is in concordance with the findings of extensive previous studies suggesting that CXCR6 is a marker of CD8^+^ T-cell activation and memory (27). Furthermore, differential expression analysis revealed increased proportions and degrees of expression of immunosuppression-related genes, such as RGS1, in CXCR6-positive T cells (**Figure 1B**, *left panel*). Examination of CXCL16-positive myeloid cells revealed enrichment of well-known immunosuppressive markers/phenotypes (C5Ar1 and CXCR4) (28–31) as well as inflammatory myeloid cells (CD93 and HLA-DPB1) (32–35), suggesting increased pleiotropic RNA expression in these subsets of GBM cells (**Figure 1B**, *right panel*).

We next sought to determine the spatial distribution of CXCL16 and CXCR6 expression in newly diagnosed and recurrent GBM patient samples using multiplexed immunohistochemistry (**Figures 1D, E**). Representative images of the multiplexed immunohistochemistry results can be found in **Supplementary Figure S1**. These samples are distinct from the scRNA-seq datasets and came from their own cohort. Interestingly, we observed a significant positive correlation between the amount of CD3^+^ T-cell infiltration and the number of CXCL16^+^ CD163^+^ immunosuppressive myeloid cells (**Figure 1D**). This was also observed when looking only at the CD8^+^CXCR6^+^ T-cell subsets (**Figure 1D**, *right panel*). Analysis of myeloid compartments revealed that the number of CXCL16^+^CD163^+^ cells was significantly positively correlated with the number of CD8+ T cells (p=0.031; **Figure 1E**, *left panel*), whereas there was a trend toward positivity in TMEM119^+^CXCL16^+^ microglia (p=0.12; **Figure 1E**, *right panel*). Importantly, when examining the total infiltration of CD163^+^ macrophages and TMEM119^+^ microglia, there was no significant correlation with CD3^+^ T-cell infiltration (**Supplementary Figure S1B**). These data suggest that there is a correlation between the presence of CXCL16^+^ myeloid cells and T-cell infiltration in GBM.

To study these interactions in our preclinical tumor models, we next sought to determine whether mouse models of GBM recapitulate these phenotypes (**Figures 1F–H**). To achieve this goal, we performed single-cell RNA sequencing (scRNA-seq) analysis of immune infiltrates and tumor cells from CT2A tumor-bearing mice. Similarly to human GBM infiltrates, CXCL16 and CXCR6 expression was restricted to myeloid cells and T cells, respectively (**Figures 1F, G**). We also plotted the data as the percentage of patients positive for CXCL16 and CXCR6 in each SingleR-defined cluster (**Supplementary Figure S2A**). Differential expression analysis of CXCR6 in the T-cell compartment in murine models also revealed preferential expression of CD8a, suggesting that its expression is restricted to CD8^+^ T cells (**Figure 1H**). Furthermore, analysis of typical activation/exhaustion markers on T cells, such as CTL4, LAG3, TIM3, and CD160, revealed only CXCR6^+^ infiltrates (**Supplementary Figure S2B**). The study revealed that CXCR6 expression is associated with activated and exhausted T cells, especially CD8^+^ T cells, while CXCL16 expression in myeloid cells is correlated with immunosuppressive phenotypes in both mouse and human GBM datasets, indicating a potential role for CXCR6 in modulating T-cell infiltration and function in GBM.

### CXCL16 is expressed on the surface of immunosuppressive myeloid cells

**Figure 2:**
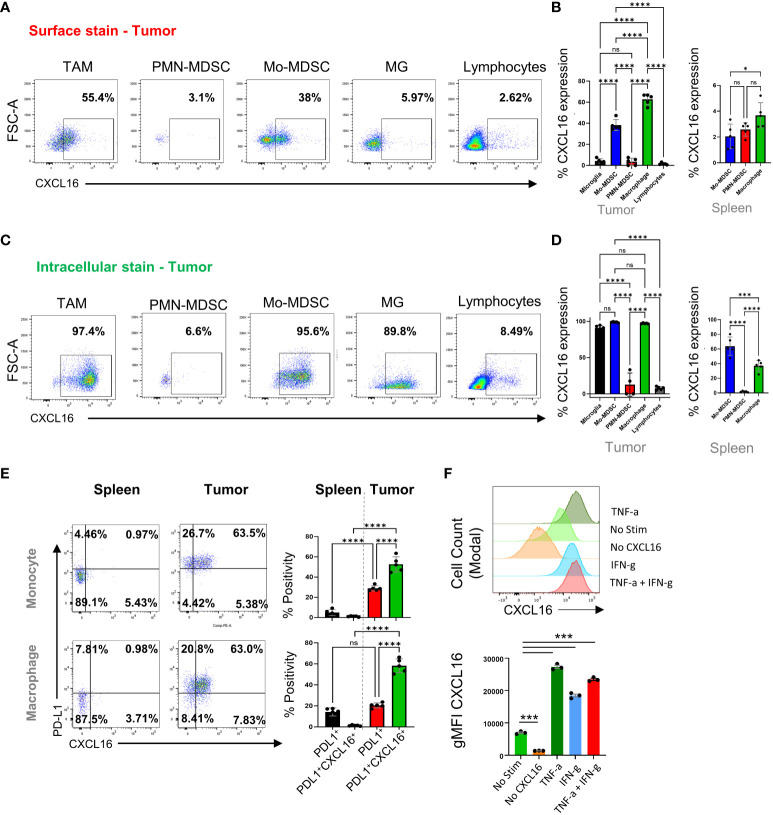
Characterization of the CXCL16/CXCR6 axis in tumor-infiltrating immune cells. In **(A, C)**, we analyzed immune cells isolated from mouse tumors by flow cytometry 14 days after tumor implantation. **(B, D)** are from n=5, showing the surface and intracellular levels of CXCL16 per cell type in tumors and spleens. Further analysis of PD-L1 and CXCL16 coexpression in tumor-associated macrophages and monocytes in the spleen and tumor tissue are shown in **(E)** and were analyzed by flow cytometry. In **(F)**, we treated TAMCs under different conditions and analyzed CXCL16 expression by flow cytometry. The significance of differences in **(B, D–F)** was analyzed by one-way ANOVA; ****p<0.0001. ns = p>0.05, *p<=0.05, ***p<=0.001.

CXCL16 has transmembrane and soluble forms that can control the migration and function of CXCR6^+^ cells. We used flow cytometry to validate how different immune cell populations express CXCL16 in CT2A tumor-bearing mice (**Figure 2**). Two weeks after tumor injection, we isolated the brains and performed surface and intracellular staining for flow cytometry. The data revealed that the surface expression of CXCL16 was restricted to the TAM and monocytic-MDSC populations (62.7 ± 2.3 and 38.2 ± 2.3%, respectively). In contrast, microglia and PMN-MDSCs did not exhibit surface expression (4.1 ± 0.9 and 3.5 ± 1.5% CXCL16 positivity, respectively) (**Figures 2A, B**). Conversely, intracellular CXCL16 was detected in TAMs, M-MDSCs, and microglia (**Figures 2C, D**), suggesting that surface expression is not uniform on the surface of myeloid cells. When immunophenotyping tumor-associated myeloid cells (TAMs) in brain tumors, four broad categories are commonly accepted: CD45^int^CD11b^int^ refers to microglia, and the rest are CD45^+^CD11b+ populations. In this gate, LY6G^-^C^-^ cells (also called Gr1-) are considered mature cells, so they are referred to as tumor-associated macrophages (TAMs). Those that were Ly6C^+^Ly7G^lo^ were considered monocytic-MDSCs, and those that were Ly6C^-^Ly6G^+^ were considered polymorphonuclear-MDSCs (refer to the following articles for gating references (9, 36–38)). We have included the gating strategy for these populations in **Supplementary Figure S3**.

On the cell surface, CXCL16 was significantly coexpressed with PD-L1 on macrophages and in monocyte-MDCS populations compared to PD-L1 alone (which was not observed in the periphery), suggesting that surface-bound CXCL16 expression is regulated by inflammation, as PD-L1 is known to be involved (39) (**Figure 2E**). The gating strategy for comparing the surface expression of CXCL16 in different myeloid cell subsets (tumor versus spleen) is shown in **Supplementary Figure S3B**. Supporting this notion, treatment with interferon or TNF dramatically upregulated surface-bound CXCL16 expression on *in vitro*-generated TAMCs (**Figure 2F**). This finding is consistent with a previous report that inflammatory signals upregulate surface CXCL16 expression and mediate firm adhesion to CXCR6^+^ cells (40). Interestingly, CXCL16 upregulation did not occur in association with the typical “M2” polarizing cytokine IL-4 (**Supplementary Figure S4**). These data demonstrated that the upregulation of CXCL16 is stimulated in response to inflammatory signals, a mechanism involved in regulating various immunosuppressive programs, including PD-L1, IDO, and Treg infiltration (39, 41, 42).

### CXCR6 promotes T-cell infiltration into GBM

**Figure 3:**
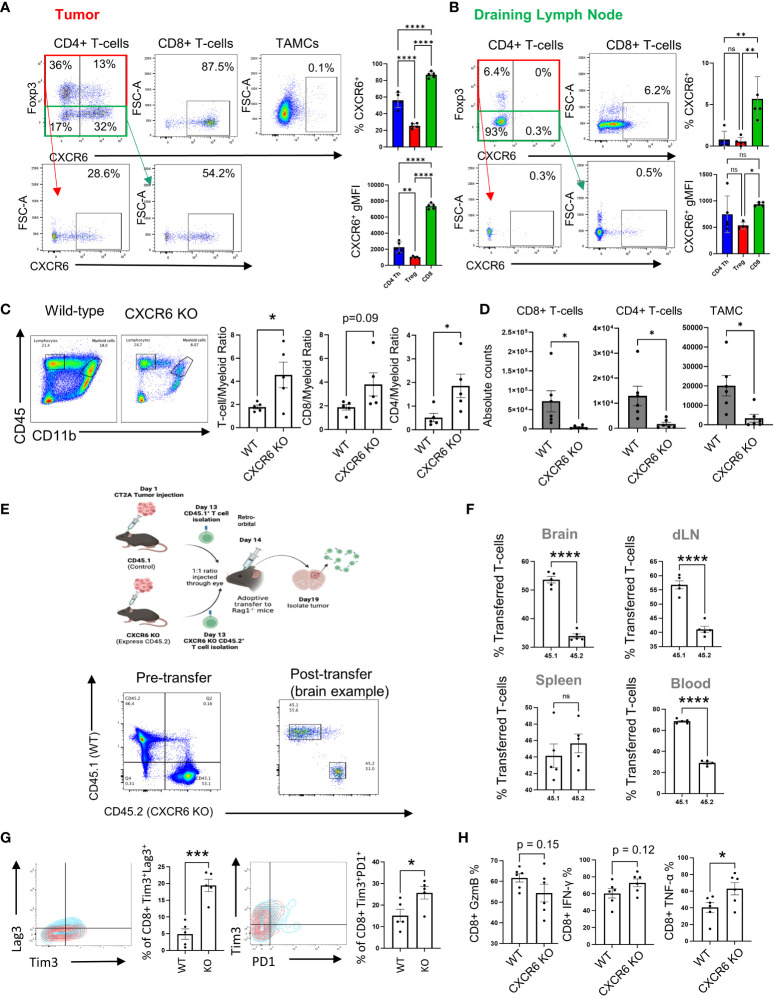
CXCR6 affects CD8^+^ T-cell migration and functions in tumors. **(A, B)** CD4^+^ and CD8^+^ T cells and TAMCs from tumors and draining lymph nodes (n=5) were isolated and analyzed by flow cytometry 14 days after tumor injection. In **(C)**, the ratio of infiltrating lymphocytes to myeloid cells was analyzed by flow cytometry. The absolute numbers of infiltrating CD4+ and CD8+ T cells and the total number of TAMCs are shown in **(D)** and were analyzed via flow cytometry. In **(E–H)**, naïve CD8+ T cells from control and CXCR6 KO mice were adoptively transferred and injected at a 1:1 ratio i.v. 5 days later. Tissues were harvested for flow cytometry analysis. Before and after the transfer, the percentages of CD45.1+ versus CD45.2+ CD8^+^ T cells were analyzed via flow cytometry (**E**, *bottom panel*). The data shown in **(F)** are from n=5, and the percentages of transferred CD8^+^ T cells in the organs were analyzed by flow cytometry. The expression of exhaustion markers (PD-1, Lag3, and Tim3) in CXCR6-deficient CD8^+^ T cells shown in **(G)** was analyzed via flow cytometry after the adoptive transfer experiment. In **(H)**, flow cytometry was used to measure the inflammatory response of GzmB/IFN-γ/TNF-α-expressing tumor-isolated WT and CXCR6 KO CD8^+^ T cells 14 days after injection. Significance in **(A, B)** was analyzed by one-way ANOVA; **(C–H)** were analyzed by Student’s t test, *p<=0.05, **p<=0.01, ***p<=0.001, ****p<=0.0001.

Examination of the effect of the CXCR6^+^ phenotype on T-cell subpopulations revealed that CD8^+^ T cells expressed more CXCR6 than did CD4^+^ T effector and Treg cells in tumors (86.4 ± 1.7 in CD8^+ cells^, 67.9 ± 2.6 in CD4^+^ T effector cells, and 41.3 ± 1.5 in Tregs; p<0.001 for all comparisons) (**Figure 3A**). This finding agrees with the single-cell data suggesting that CD8+ T cells predominantly express CXCR6 and with previous publications suggesting that CD8^+^ T cells are the predominant subset expressing this marker (25, 43–45). Similarly, compared with that in tumors, expression of CXCR6 in T cells in draining lymph nodes was largely absent, which suggested that the CXCL16/CXCR6 axis might be relevant only after T-cell activation (5.3 ± 1.1 in CD8^+ cells^, 1.4 ± 0.6 in CD4^+^ T effector cells, and 1.3 ± 0.6 in Tregs; p<0.01 for CD8^+^ cells compared with both CD4^+^ subsets) (**Figure 3B**). Indeed, our *in vitro* data also support that CXCR6 expression is significantly increased after TCR stimulation in CD8^+^ T cells, whereas CD4^+^ T cells exhibit minimal upregulation of CXCR6 (**Supplementary Figure S5**). Importantly, FMO controls for CXCR6 were used to determine the accuracy of the staining. The top panel shows the results for each panel.

Knowing how the CXCL16/CXCR6 axis is expressed by myeloid cells and T cells in tumors, the next step was to understand how this axis influences the composition of immune cell populations in the tumor microenvironment. To examine the role of the CXCR6/CXCL16 axis in GBM progression, we implanted CT2A tumors into WT or CXCR6 KO mice, and after 14 days of engraftment, flow cytometry analysis was performed (**Figures 3C, D**). The first observation was that there was an increased T cell: myeloid cell ratio in the CXCR6 knockout tumor microenvironment, which is suggestive of a more inflammatory tumor (1.8 ± 0.2 *vs*. 4.6 ± 1.1 T-cell/TAMC ratio, 2.6 ± 0.5 *vs*. 5.0 ± 1.2 CD8/TAMC ratio, and 0.5 ± 0.2 *vs*. 1.9 ± 0.5 CD4/TAMC ratio in the control versus CXCR6 KO, respectively) (**Figure 3C**). When examining absolute cell counts, there were significantly fewer immune infiltrates of both T-cell and myeloid populations into the tumors of CXCR6 knockout mice (6.8x10^4^ ± 3.3x10^3^ *vs*. 4.7x10^4^ ± 1.9x10^3^ CD8^+^ T cells, 1.3x10^4^ ± 4.8x10^3^ *vs*. 1.9x10^3^ ± 8.3x10^2^ CD4^+^ T cells, and 2.0x10^4^ ± 6.4x10^3^ *vs*. 4.0x10^3^ ± 2.4x10^3^ TAMCs, absolute counts in control versus CXCR6 KO tumor-bearing mice, respectively) (**Figure 3D**). These data suggest that CXCR6 is important for T-cell recruitment to tumors.

To determine whether T-cell migration is regulated by CXCR6, we used a transwell system in which increasing concentrations of CXCL16 were used as a chemoattractant (**Supplementary Figure S6**). While FBS was able to rapidly induce T-cell migration across both the 3 µM and 5 µM inserts, recombinant CXCL16 had no effect. This finding suggested that, alone, CXCL16 cannot promote robust T-cell migration. This finding is consistent with previous *in vitro* work identifying it as a weak chemoattractant (46). However, these *in vitro* assays do not recapitulate the myriad of signals that occur *in vivo*; therefore, we performed an adoptive transfer experiment to evaluate competition between wild-type and CXCR6 knockout T cells to determine how CXCR6 influences T-cell migration to tumors (**Figures 3E, F**). To investigate the underlying mechanism of migration, we isolated T cells from donor mice two weeks after tumor injection, with control T cells labeled 45.1 and CXCR6 knockout T cells labeled CD45.2. After donor T cells were activated by CD3/CD28 Dynabeads, we intravenously transferred cells at a 1:1 ratio to tumor-bearing Rag^-/-^ mice, ensuring a confirmed ratio before adoptive transfer (**Figure 3E**). After 5 days, we isolated the tumor and peripheral tissues for analysis via flow cytometry. We examined the localization of the transferred T cells in different organs. We found that in the spleen, there was a similar number of control and knockout CD8 T cells (44.1 ± 1.4% *vs*. 45.6 ± 1.1% in Control compared to CXCR6 KO; ns) but significantly fewer CXCR6 knockout CD8 T cells in the brain (53.6 ± 1.2% in Control *vs*. 34.0 ± 0.8% in CXCR6 KO; p<0.001), draining lymph nodes (56.8 ± 1.4% in control *vs*. 41.1 ± 1.1% in CXCR6 KO; p<0.001), and blood (68.6 ± 0.9% in Control *vs*. 29.0 ± 1.0% in CXCR6 KO; p<0.001), showing that this chemokine receptor promotes CD8 T-cell infiltration to infiltrate tumors (**Figure 3F**).

We also inspected whether CXCR6 can influence T-cell immunosuppression by affecting the expression of activation and exhaustion markers after adoptive transfer. The data revealed increased TIM3^+^LAG3^+^ (4.9 ± 1.6% in the control *vs* 19.5 ± 1.9% in the CXCR6 KO; p<0.001) and PD1^+^TIM3^+^ (15.2 ± 2.9% in the control *vs* 25.7 ± 2.9% in the CXCR6 KO; p<0.05) coexpressing cells among the CXCR6-deficient CD8^+^ T cells (**Figure 3G**). Therefore, these data suggest that while CXCR6 is important for T-cell trafficking to tumors, it may prevent T-cell activity within tumors. Supporting this notion, control and CXCR6 KO tumors were implanted, and T-cell cytokine production was measured via flow cytometry (**Figure 3H**). TNFα was expressed at significantly greater levels in the CXCR6 knockout CD8^+^ T cells (60.6 ± 6.4% in the control *vs*. 76.9 ± 7.0% in the CXCR6 KO; p<0.05), while Granzyme B (61.4 ± 2.4% in the control *vs*. 53.3 ± 5.1% in the CXCR6 KO; ns) and IFNγ (57.1 ± 5.3% in the control *vs*. 71.4 ± 5.5% in the CXCR6 KO; ns) were trending toward an increase in CXCR6 KO T cells but not significantly. The results of this work suggest that there might be divergent roles for CXCR6/CXCL16 in antitumor immunity.

### Intratumoral myeloid-T-cell interactions are driven by CXCR6/CXCL16 and promote immunosuppression

**Figure 4:**
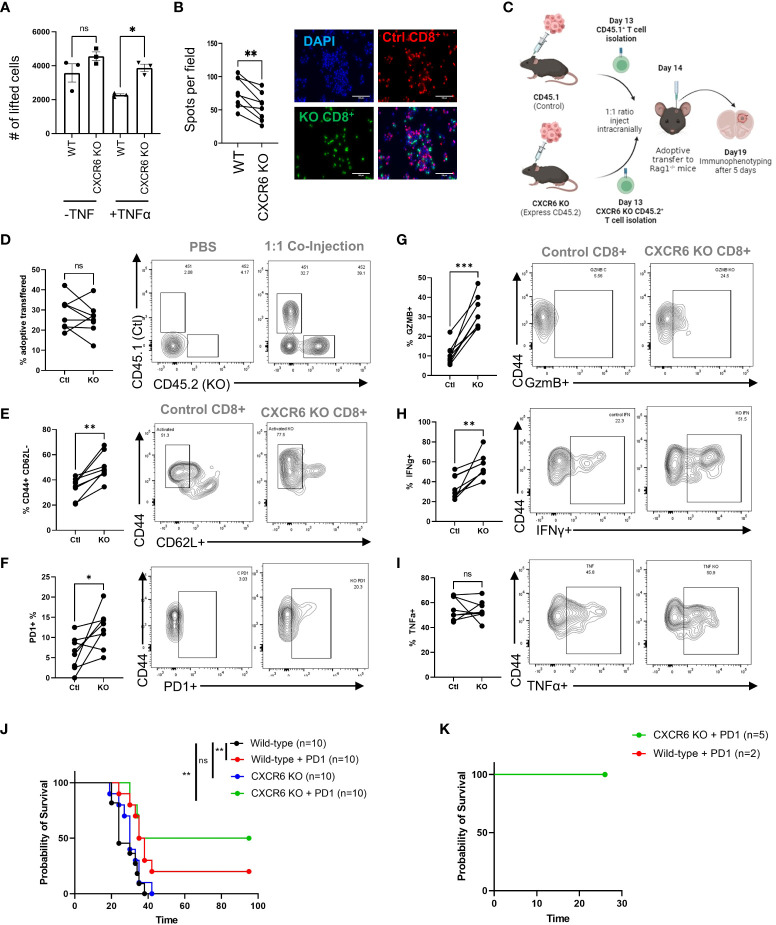
CXCR6 deficiency prevents CD8^+^ T-cell–TAMC interactions and increases CD8^+^ T-cell activity and survival. **(A)** CD8^+^ T cells from control or CXCR6 KO mice were isolated, activated *in vitro*, labeled with a fluorescent-tracker dye, and then cocultured at a 1:1 ratio with tumor-associated myeloid cells (TAMCs). After 90 minutes, the slides were washed and analyzed via epifluorescence microscopy. The data shown in **(A, B)** are from 4 replicates, and two images per slide were analyzed. A paired Student’s t test was performed, and *p*<0.001 = **. **(C)** Tumors were isolated on the 19th day after the adoptive transfer experiment, and the data in **(D)** show the CD45.1 and CD45.2 ratios in tumors without and after transfer, as analyzed by flow cytometry. In **(G–I)**, flow cytometry analysis of the coexpression of CD44 with CD62L, PD1, GzmB, INF-γ, and TNF-α in CD45.1^+^ or CD45.2^+^ CD8^+^ T cells is shown. The CXCR6 knockout group treated with PD-1 blockade achieved 50% survival. **(J)**. Median survival: control = 24 days, control + PD-1 blockade = 36.5 days, CXCR6 KO = 30 days, CXCR6 KO + PD-1 blockade = 65 days; *p*= 0.002 = ** Log-rank test). **(K)** Surviving mice were rechallenged via tumor implantation. Tumor rejection in both groups represented the generation of immunologic memory against tumor antigens. Significance in **(D–I)** was analyzed by paired Student’s t test; *p<=0.05, **p<=0.01, ***p<=0.001.

As our previous data clearly indicated that immunosuppressive myeloid cells express surface-bound CXCL16, we reasoned that even though CXCR6 is needed for T cells to infiltrate GBM tissue, once in the tissue, the CXCR6/CXCL16 interaction may foster immunosuppression in T cells by fostering interactions between T cells and immunosuppressive TAMCs. To address this possibility, we performed two types of adhesion assays previously used to evaluate CXCL16/CXCR6 interactions (20) (**Figures 4A, B**).

Inflammation induces surface expression of CXCL16 in myeloid cells (**Figure 2A**). Pretreatment of TAMCs with TNF-ɑ resulted in decreased recovery of wild-type CD8^+^ T cells, suggesting that CD8^+^ T cells were more adherent, while CXCL16 was highly expressed. Surprisingly, the number of recovered CXCR6 knockout CD8^+^ T cells was significantly greater than that of control T cells in the TNF-ɑ treatment group (2.3x10^3^ ± 5.4x10^1^ cells in the control *vs* 3.9x10^3^ ± 1.7x10^2^ in the CXCR6 KO; p<0.05), suggesting that the TNF-ɑ-mediated increase in CXCL16 promotes T-cell/myeloid cell interactions (**Figure 4A**). In addition, we designed another type of adhesion assay to visualize nonlifted CD8^+^ T cells. In this assay, both control and CXCR6 KO T cells were labeled with fluorescent dyes and plated together at a 2:1 ratio into the same culture of TAMCs, after which the nonadherent cells were vigorously washed off the plate (**Figure 4B**). According to the immunofluorescence images, we observed fewer CXCR6 knockout CD8^+^ T cells in the field, suggesting that more receptor knockout T cells were lifted, suggesting that, without CXCR6, the CD8^+^ T cells interact less with TAMCs *in vitro* (76.25 ± 8.5 control cells per field *vs*. 54.5 ± 7.9 CXCR6 KO T cells per field; paired t test, p<0.01). These data suggest that if we bypass the deficiency in T-cell recruitment in CXCR6 KO T cells, CXCR6 KO T cells may be more functional.

To address this possibility, WT CD45.1 CD8^+^ T cells and CD45.2 CXCR6 KO T cells were activated for 24 hours before being injected at a 1:1 ratio into RAG-1KO mice harboring CT-2A brain tumors (**Figure 4C**). Five days after coinjection, immune phenotypes were assessed in the tumors of the mice. There was no obvious difference in the viability of T cells from these tumors, as the percentages of CD45.1 WT and CD45.2 KO cells in tumors were not significantly different (28.4 ± 3.0% in controls *vs*. 25.8 ± 2.9% in KO, ns) (**Figure 4D**). Conversely, several markers of T-cell activation and functionality were significantly increased in CXCR6 KO T cells. The percentage of T cells with an activated/memory phenotype (CD44^+^CD62L^-^) was significantly greater in CXCR6 KO CD8^+^ T cells than in CD45.1 WT T cells (33.9 ± 3.0% in controls *vs*. 50.3 ± 3.8% in KO, p=0.0015) (**Figure 4E**). Similarly, CXCR6KO T cells had increased expression of PD1 (6.1 ± 1.5% in controls *vs*. 12.2 ± 1.7% in KO, p=0.03), Granzyme B (10.7 ± 1.9% in controls *vs*. 32.2 ± 2.9% in KO, p<0.001), and Interferon-γ (34.3 ± 4.3% in controls *vs*. 56.5 ± 4.2% in KO, p<0.001) (**Figures 4F–H**). There was no change in TNFα expression (**Figure 4I**), which may be reflective of its role as an early activation marker (47). Finally, to determine whether CXCR6 deficiency influences antitumor responses, we implanted control or CXCR6 KO cells with CT-2A and administered an anti-PD1 immune checkpoint blockade (**Figure 4J**). While only 20% of the WT mice responded to immunotherapy, 50% of the CXCR6 KO mice rejected the tumors. Furthermore, reimplantation in the contralateral hemisphere of these mice resulted in no tumor formation, indicating that immunological memory had formed in these mice (**Figure 4K**). The results of this study indicate that the role of the CXCR6/CXCL16 axis in GBM differs depending on where the immune cells are located.

## Discussion

In our study, we focused on revealing the intricate role of the CXCR6/CXCL16 axis in T-cell myeloid interactions within GBM tissues. Our findings reveal a nuanced picture of how this axis contributes to both immune activation and immunosuppression, shedding light on its context-dependent functions. Importantly, we observed distinct patterns of CXCL16 expression, with TAMCs primarily displaying surface expression, while microglia release CXCL16 as a cytokine. One key insight from our research is the pivotal role of CXCR6 in T-cell activation and initial migration into tumor tissues. This finding suggested that the CXCL16-CXCR6 axis serves as an essential bridge facilitating the early stages of T-cell immune responses, facilitating T-cell infiltration into GBM.

Indeed, previous studies have indicated that CXCR6 plays a role in T-cell homing to tissues, particularly the liver. For example, Muthuswamy et al. demonstrated that CXCR6^+^ is essential for the retention of memory T cells in ovarian tumor tissues (44). In another study, CXCR6^+^ promoted T-cell trafficking to livers infected with Listeria spp (48). This finding was similar to that of another study in which CXCR6 was found to be critical for CD8^+^ memory in the liver in response to infections (49). Our data support these observations by showing that there is reduced T-cell infiltration into brain tumor tissue. This finding is consistent with a very recent study showing that CXCR6 is essential for CD8^+^ T-cell residency in the CNS and that it acts to prevent Alzheimer’s disease pathology (45).

However, once T cells are situated within the tumor, this axis appears to pivot toward promoting immunosuppression, as indicated by the observed T-cell dysfunction in our adoptive transfer experiments. Furthermore, mice deficient in CXCR6 were more responsive to anti-PD1 therapy, also suggesting that this axis promotes immunosuppression in tumors. Indeed, a single study previously examining the CXCL16/CXCR6 axis in GBM demonstrated that CXL16 expression in myeloid cells (particularly microglia) allows cells to adopt an anti-inflammatory state and that in the context of glioma, it contributes to immunosuppression (50). Furthermore, CXCR6 KO mice exhibited a significant increase in survival, indicating that this axis can drive immunosuppression (50). Our study adds to this knowledge base by examining the role of surface-bound CXCL16 in coordinating myeloid T-cell interactions within GBM tissues.

Indeed, our study showed that CXCR6^+^ KO T cells are less adherent to tumor-associated myeloid cells and are more functional when directly injected into tumor tissue. This finding is consistent with some emerging data examining the blockade of CXCL16 in breast cancer (51). In this study, the authors found that blocking CXCL16 prevented CD8^+^ T-cell infiltration into tumors and decreased PD-1 expression, which was consistent with our observations from our knockout models and adoptive transfer experiments. This group also demonstrated that the combination of PD1 and CXCL16 blockade led to increases in CD8^+^ T-cell infiltration in tumors, resulting in the promotion of antitumor immunity (51). These findings are also consistent with our data showing enhanced antitumor immunity in CXCR6 KO mice treated with anti-PD1 therapy.

This dualistic nature of the CXCL16-CXCR6 axis in GBM underscores its significant influence on antitumor immunity and provides some clarity on the opposing roles of this axis in cancer. The results of this work, especially in the context of the previous research described above, suggest that the CXCR6/CXCL16 interaction between myeloid cells and T cells does not dictate immunosuppression or immune activation but rather simply facilitates the interaction between two cells. In other words, while in the periphery, CXCR6 may be critical for T cells to interact with DCs and subsequently migrate to tissues (such as the liver or brain), when these cells interact with immunosuppressive cells, immunosuppression results.

Our scRNA-seq and spatial multiplexing data support this theory. CXCR6 is expressed by dysfunctional cells in human GBM tissues and is located more closely related to CD163^+^ macrophages, which are a well-known immunosuppressive subset of macrophages in GBM (52, 53). While CXCL16 is expressed by both infiltrative myeloid cells and microglia both transcriptionally and via multiplex IHC, only surface-bound CXCL16 was detected on immunosuppressive subsets. This protein was also expressed along with the IFN-responsive immunosuppressive marker PD-L1 (39), which suggested that surface expression is involved in reactive immunosuppression in GBM. This finding suggested that, perhaps when microglia are activated, they can also express CXCL16 on their surface, although future studies will need to address this possibility. The reason why there seems to be an overall reduction in TAMC infiltration as well as T-cell infiltration in CXCR6 KO tumor-bearing mice is not clear. It is possible that myeloid infiltration is responsive to inflammatory signals from the TME, which has been demonstrated previously in response to radiation therapy (54). Future work will need to be done to elucidate this phenomenon.

In conclusion, our study underscores the dualistic nature of the CXCL16-CXCR6 axis in GBM, where it serves as a critical mediator of both early T-cell immune responses and subsequent immunosuppression within the tumor microenvironment. While targeting this axis holds promise for therapeutic interventions in GBM, the timing and context of intervention must be carefully considered. These findings provide valuable insights into the complex interplay between myeloid cells and T cells within the GBM microenvironment, offering potential avenues for the development of more effective immunotherapies for this devastating disease.

## Limitations

It is important to acknowledge that there are several limitations in our study. Although our research strongly supports the idea of the predominant expression of CXCR6 on CD8+ T cells and the facilitation of antitumor activity during CXCR6 knockout, without extensive exploration of the implications of CD4+ T cells in the tumor microenvironment, a gap in understanding of the role of CXCR6 in CD4-mediated tumor immunity remains. Indeed, several previous studies have indicated that CXCR6 expression on CD4+ T cells can regulate inflammation (46, 55, 56) and that CXCR6 is expressed on resting memory T cells (57). Thus, future investigations should be performed to provide more comprehensive research on the impact of CXCR6 on the interaction and function of CD4+ T cells in GBM.

Moreover, our study focused mainly on the CT-2A model of GBM, suggesting that CXCR6-deficient mice respond positively to anti-PD1 therapy. These results are slightly different from previous work on this topic using the GL-261 model of GBM (50). In this study, the authors found that CXCR6 KO mice lived significantly longer than WT mice; in our model, survival benefit could be extended only in the context of immunotherapy. We suspect that this difference is due to the differences in the immunosuppressive phenotypes of these models. As studies have shown that GL-261 cells are generally more responsive to immunotherapy than CT-2A cells (5, 58), these differences may partially explain the differences between these studies. Therefore, caution must be taken when comparing our therapeutic results with those of other tumor models.

## Data availability statement

The RNA-seq data for this study can be found in the NIH bioproject repository; https://www.ncbi.nlm.nih.gov/bioproject/PRJNA909275. The scRNA-seq data can be found in the NIH bioproject repository: https://www.ncbi.nlm.nih.gov/bioproject/1029174.

## Ethics statement

The animal study was approved by Northwestern University Institutional Animal Care and Use Committee. The study was conducted in accordance with the local legislation and institutional requirements.

## Author contributions

T-YC: Formal analysis, Methodology, Writing – original draft, Writing – review & editing, Investigation. LKB: Investigation, Methodology, Supervision, Writing – original draft, Writing – review & editing, Data curation. LB: Formal analysis, Writing – original draft, Writing – review & editing, Investigation. JLK: Investigation, Methodology, Writing – original draft, Writing – review & editing. VA: Investigation, Methodology, Writing – original draft, Writing – review & editing. JS: Formal analysis, Investigation, Methodology, Writing – original draft, Writing – review & editing. A-LR: Investigation, Methodology, Writing – original draft, Writing – review & editing. SD: Formal analysis, Investigation, Methodology, Writing – original draft, Writing – review & editing. KZ: Investigation, Methodology, Writing – original draft, Writing – review & editing. YG: Investigation, Methodology, Writing – original draft, Writing – review & editing. JK: Writing – review & editing. CS: Investigation, Methodology, Writing – original draft, Writing – review & editing. HWg: Investigation, Methodology, Writing – original draft, Writing – review & editing. GV: Investigation, Methodology, Writing – original draft, Writing – review & editing. DH: Investigation, Methodology, Writing – original draft, Writing – review & editing. SW: Investigation, Methodology, Writing – original draft, Writing – review & editing. HWn: Investigation, Methodology, Writing – original draft, Writing – review & editing. AS: Resources, Supervision, Writing – review & editing. PZ: Investigation, Methodology, Supervision, Writing – original draft, Writing – review & editing. CL-C: Funding acquisition, Investigation, Methodology, Supervision, Writing – original draft, Writing – review & editing. JM: Conceptualization, Data curation, Formal analysis, Funding acquisition, Methodology, Project administration, Resources, Writing – original draft, Writing – review & editing.
