# Supplementary figures and images for: The CXCL16-CXCR6 axis in glioblastoma modulates T-cell activity in a spatiotemporal context
Source: Front Immunol. 2024 Jan 17;14:1331287. doi: 10.3389/fimmu.2023.1331287 (PMC10827847; doi:10.3389/fimmu.2023.1331287)

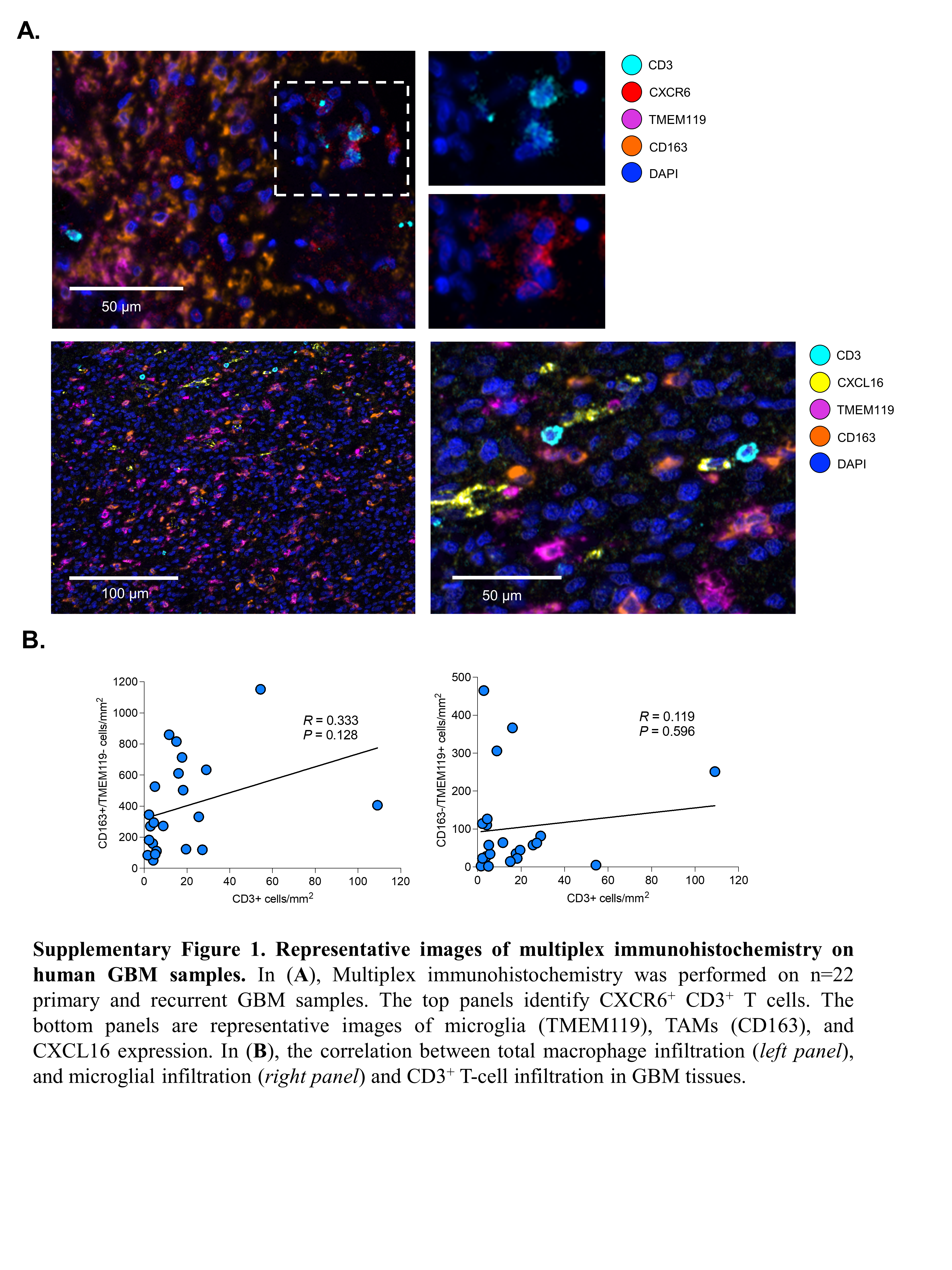

Supplement: Supplementary file 1 [file Image_1.tif]

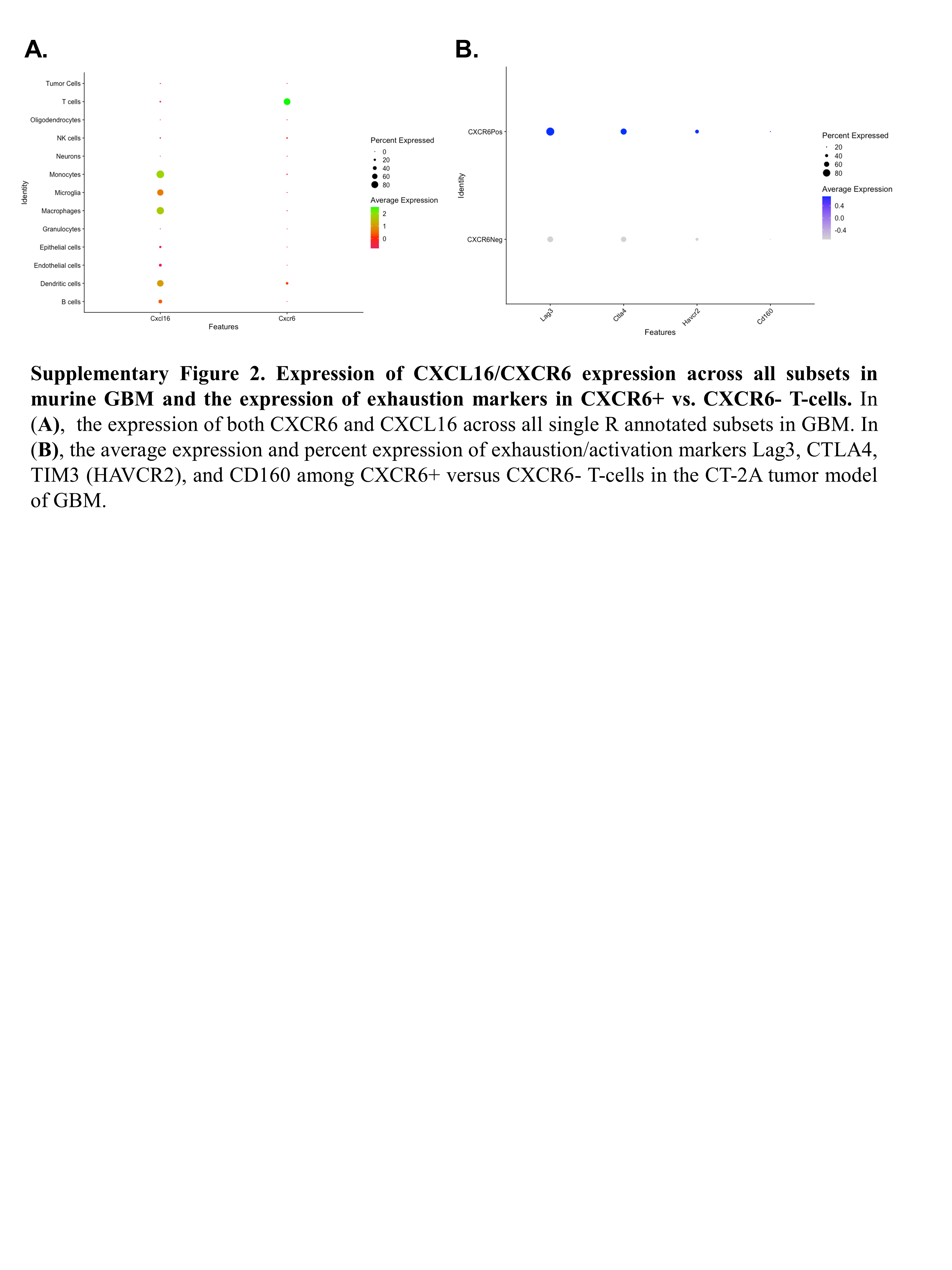

Supplement: Supplementary file 2 [file Image_2.tif]

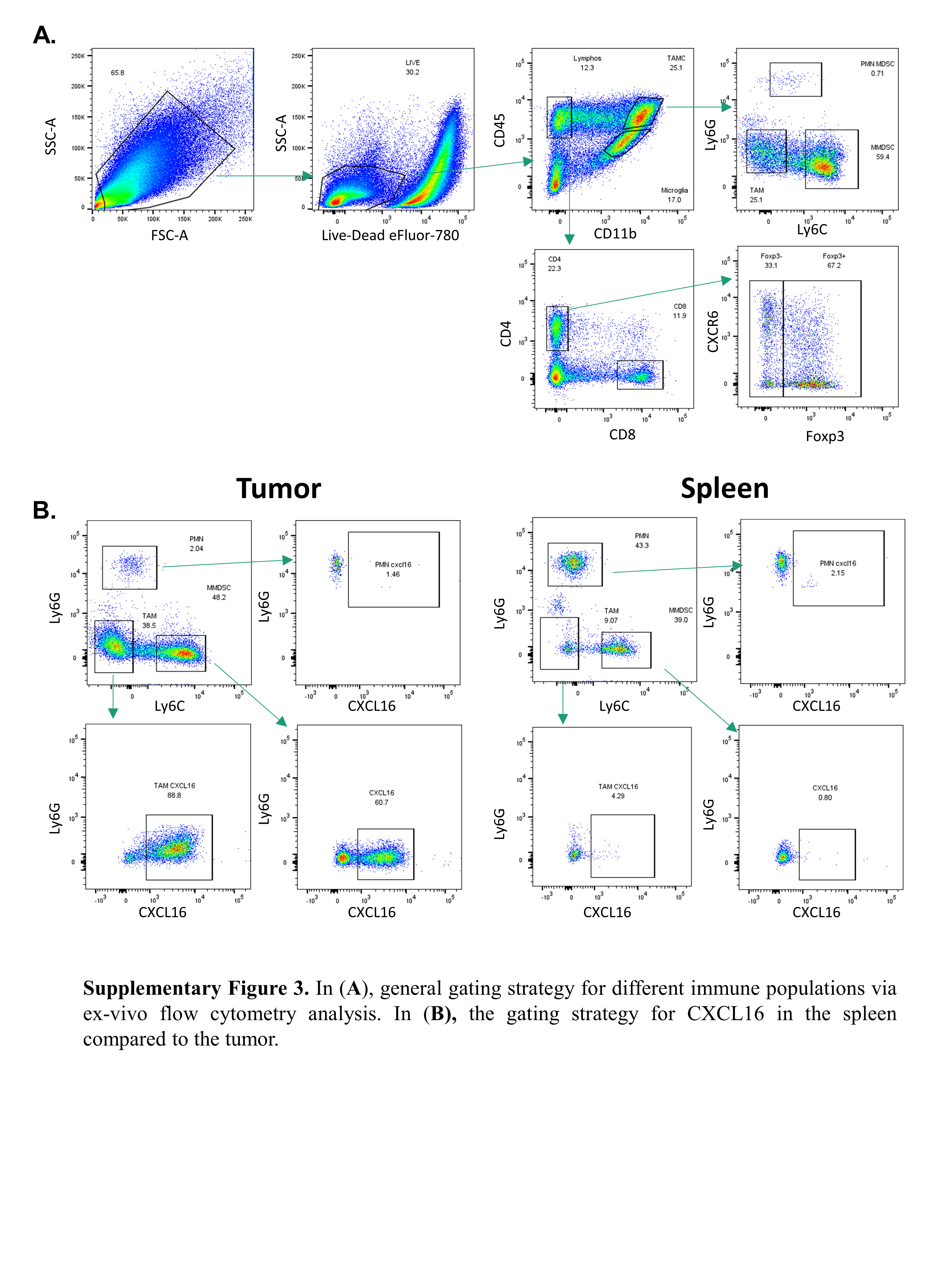

Supplement: Supplementary file 3 [file Image_3.tif]

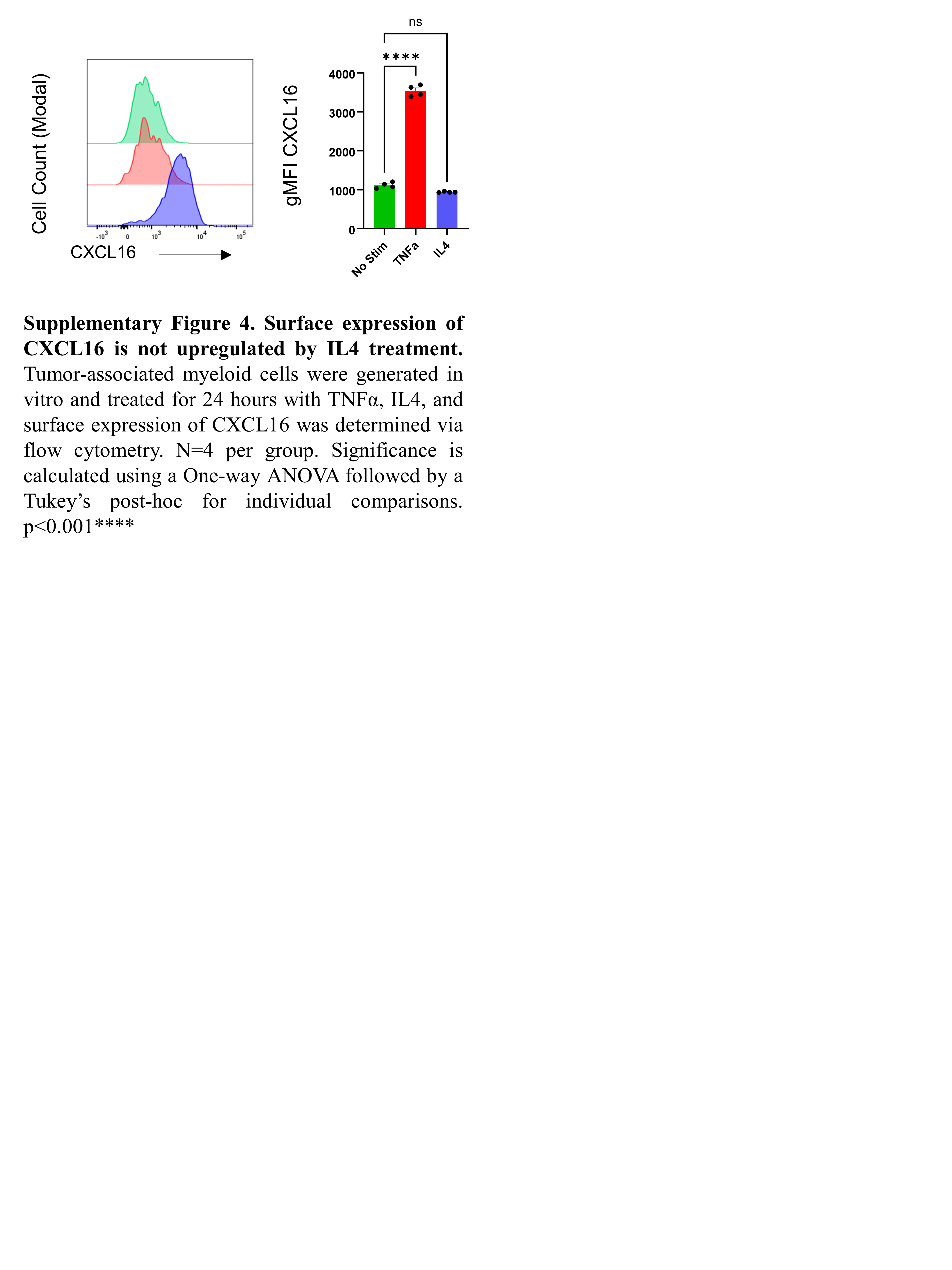

Supplement: Supplementary file 4 [file Image_4.tif]

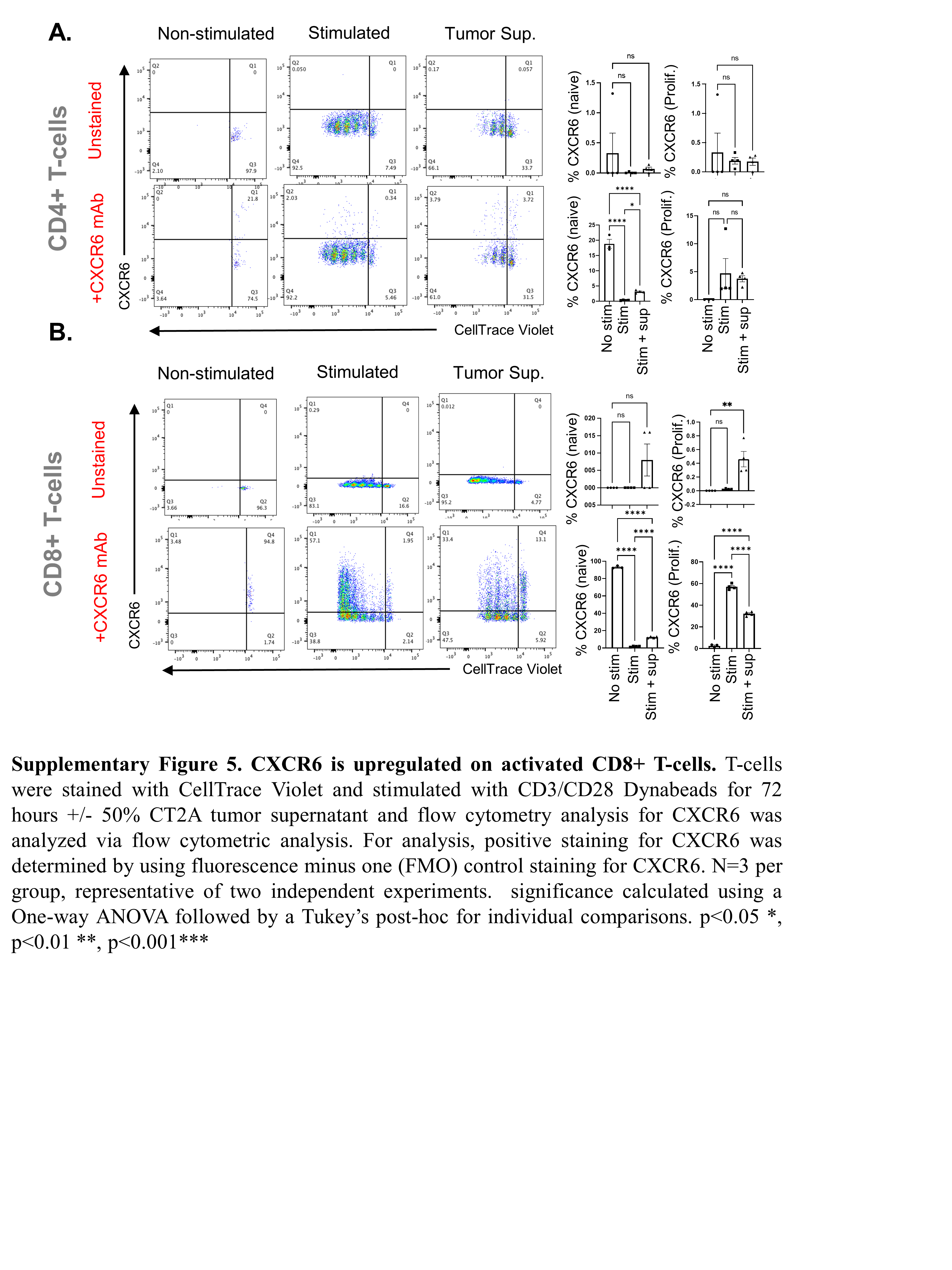

Supplement: Supplementary file 5 [file Image_5.tif]

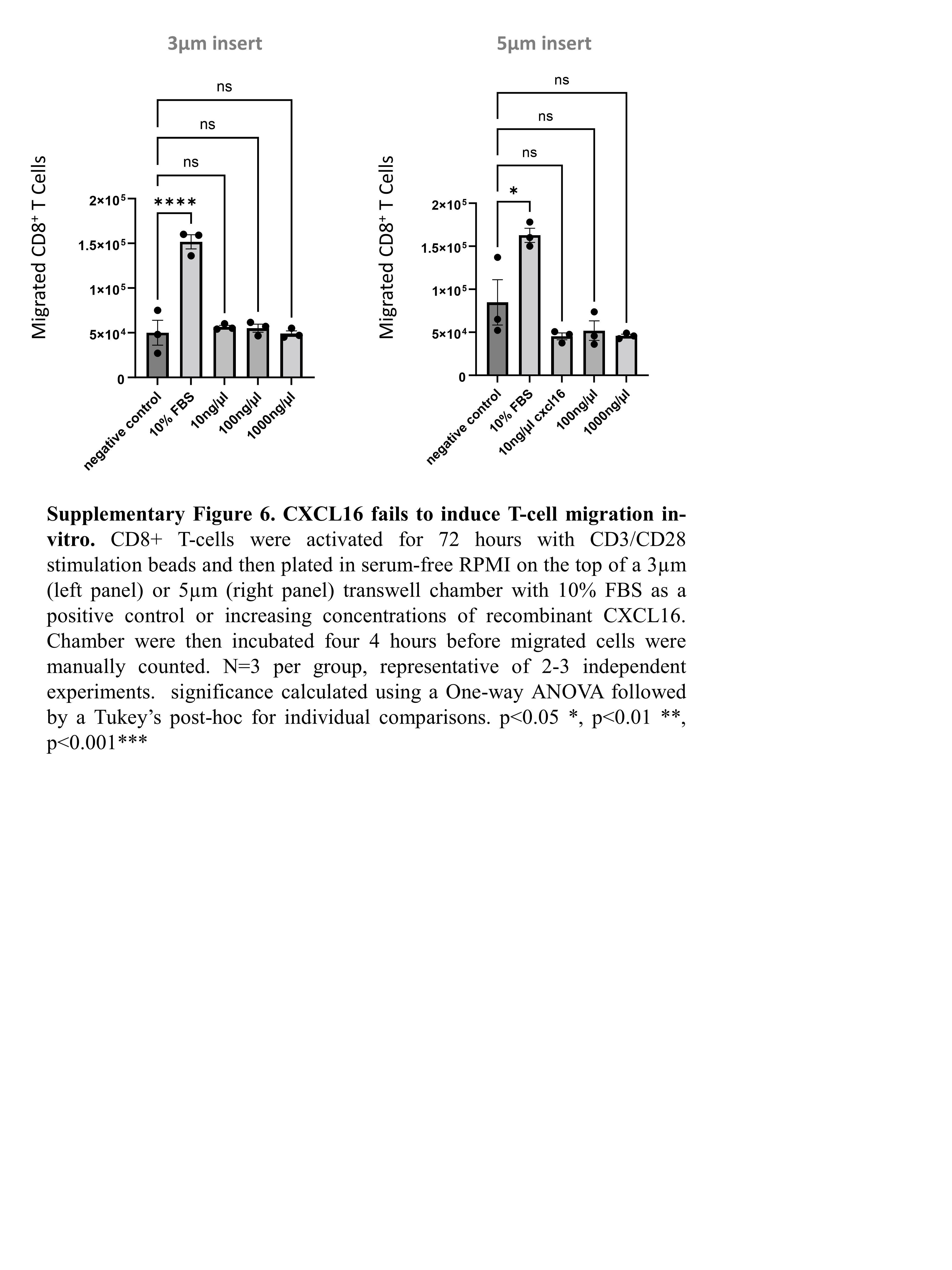

Supplement: Supplementary file 6 [file Image_6.tif]
